# Hydroxysafflor Yellow A: A Promising Therapeutic Agent for a Broad Spectrum of Diseases

**DOI:** 10.1155/2018/8259280

**Published:** 2018-09-25

**Authors:** Hui Ao, Wuwen Feng, Cheng Peng

**Affiliations:** ^1^College of Pharmacy, Chengdu University of Traditional Chinese Medicine, Chengdu 611137, China; ^2^Innovative Institute of Chinese Medicine and Pharmacy, Chengdu University of Traditional Chinese Medicine, Chengdu 611137, China

## Abstract

Hydroxysafflor yellow A (HSYA) is one of the major bioactive and water-soluble compounds isolated from Carthami Flos, the flower of safflower (*Carthamus tinctorius* L.). As a natural pigment with favorable medical use, HSYA has gained extensive attention due to broad and effective pharmacological activities since first isolation in 1993. In clinic, the safflor yellow injection which mainly contains about 80% HSYA was approved by the China State Food and Drug Administration and used to treat cardiac diseases such as angina pectoris. In basic pharmacology, HSYA has been proved to exhibit a broad spectrum of biological effects that include, but not limited to, cardiovascular effect, neuroprotection, liver and lung protection, antitumor activity, metabolism regulation, and endothelium cell protection. Although a great number of studies have been carried out to prove the pharmacological effects and corresponding mechanisms of HYSA, a systemic review of HYSA has not yet been seen. Here, we provide a comprehensive summarization of the pharmacological effects of HYSA. Together with special attention to mechanisms of actions, this review can serve as the basis for further researches and developments of this medicinal compound.

## 1. Instruction

**Figure 1:**
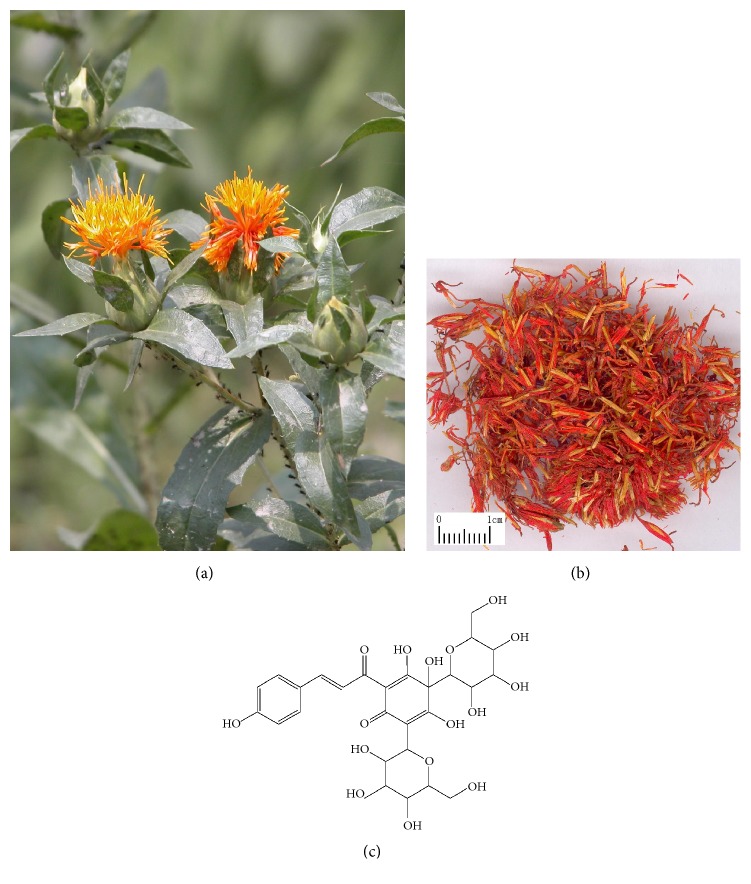
Hydroxysafflor yellow A (HYSA) and its sources.

*Carthamus tinctorius* L. (Figure 1(a)), also named safflower, belongs to the genus Carthamus family Compositae. As a multipurpose cash crop in agriculture, industry, and medicine, it is cultivated for its seeds, meals, and flowers. In terms of medical use, safflower is widely applied in East Asia especially in China [1].

Carthami Flos (Figure 1(b)), also named Honghua in China, is the dried floret of safflower and known as a blood stasis promoting herb. Golden Chamber Synopsis (Jin Gui Yao Nüe) by Zhang Zhongjing in the Han Danasty deemed the decoction of Carthami Flos as an effective remedy for gynaecological problems. Carthami Flos was firstly introduced as a medicinal herb in Annotation of Materia Medica (Xin Xiu Ben Cao) of the Tang Dynasty for the treatment of lockjaw, hemonode, and postpartum illness. Since then, the extracts of Carthami Flos have been extensively applied to treat several diseases such as cardiovascular and cerebrovascular disorders caused by blood stasis.

(a)* Carthamus tinctorius* L. (Safflower). (b) Carthami Flos (the dried flower of* Carthamus tinctorius* L.). (c) Hydroxysafflor yellow A (the main effective compound of Carthami Flos). Hydroxysafflor yellow A (HYSA, Figure 1(c)) is a water-soluble compound mainly responsible for the medicinal activities of Carthami Flos. It was firstly separated from* Carthamus tinctorius* L. by Meselhy* et al*. in 1993 [2] and is regarded as one of the standard components for quality control of Carthami Flos according to the Chinese Pharmacopoeia, because of its rich abundance and strong activities [3]. In 2005, the Safflor yellow injection which contained 45 mg HSYA per 50 mg was approved as a novel cardiovascular drug by China State Food and Drug Administration and began to be widely used for treatment of cardiac diseases such as angina pectoris. The systematic evaluations demonstrated that this injection was significantly effective for both angina pectoris and cerebral infarction [4, 5]. Apart from the effect on cardiovascular disorders, HYSA showed neuroprotection, anticancer properties, and metabolism regulation as well as liver, lung, and endothelium cell protection. In this review, we managed to give a comprehensive review and analysis of the pharmacological properties of HYSA, supporting the potential application of HYSA in clinic.

## 2. Pharmacology

### 2.1. Cardiovascular Effects

In clinic, products containing HYSA are mostly used for treatment of cardiovascular diseases. The therapeutic effect of HYSA on cardiovascular diseases is related to its anticoagulant effect, antimyocardial ischemia activity, vasorelaxative effect, etc.

#### 2.1.1. Anticoagulant Effect

The anticoagulant action of HYSA was investigated mainly* in vitro*. HYSA could elicit suppressive effects on thrombosis formation in the normal rats and rabbit platelet aggregation induced by adenosine diphosphate (ADP) and platelet activating factor (PAF) as well as rabbit blood viscosity* ex vivo* [6, 7]. It could also prolong prothrombin time (PT) of rat plasma and recalcification time (RT) of rabbit plasma* in vitro* [8].

#### 2.1.2. Effect on Myocardial Ischemia

As an antiangina drug, the cardiac protection of HYSA has been observed* in vivo* and* in vitro*. HSYA could reverse the haemodynamic alteration, enhance the survival rate, alleviate the myocardial damage, and promote the angiogenesis in the ischemic myocardium by enhancing expression of nucleolin and thus upregulating expressions of vascular endothelial growth factor A (VEGF-A) and matrix metallopeptidase 9 (MMP-9) in the myocardial ischemia (MI) rats induced by occlusion of left anterior descending coronary artery (LAD) [9]. In addition, HYSA could also lower the levels of cardiac troponin I (cTnI) and 8-hydroxy-2′-deoxyguanosine (8-OHdG) in the MI mice induced by LAD [10].* In vitro*, HYSA could relieve the nuclear morphological changes and levels of malondialdehyde (MDA) and reactive oxygen species (ROS), lower the activities of creatine kinase-MB (CK-MB) and lactate dehydrogenase (LDH), and increase mitochondrial membrane potential (MMP) and expressions of peroxisome proliferator-activated receptor gamma coactivator-1*α* (PGC-1*α*) and nuclear factor erythroid 2-related factor 2 (Nrf2) in H9C2 cells suffered from oxygen-glucose deprivation (OGD) [11].

Inhibition of mitochondrial permeability transition pore (MPTP) opening can protect heart from ischemia/reperfusion (I/R) injury. The protective effect of HSYA on the myocardial ischemia/reperfusion (MI/R) rats and the ventricular myocytes isolated from those animals could be attenuated by a MPTP opener called atractyloside and a restrainer of nitric oxide synthase (NOS) named L-NAME [12]. Meanwhile, in the isolated cardiac myocytes stimulated by anoxia/reoxygenation or ionomycin, HYSA increased rod shape cells in the closed MPTP condition and decreased round cells with open MPTP [13]. Another two targets of HYSA against MI or MI/R damage are hemeoxygenase-1 (HO-1) and hemeoxygenase-2 (HO-2). HSYA was able to promote neovascularization and cardiac function recovery* in vivo* and* in vitro* by acting through the HO-1/VEGF-A/stromal cell derived factor-1 (SDF-1*α*) cascade [14]. In hypoxia/reperfusion- (H/R-) induced H9C2 cells, HYSA exhibited antiapoptotic and antioxidative effects by mediating the protein kinase B (Akt)/Nrf2/HO-1 signaling pathway [10, 15]. Jia* et al*. [16] also reported that HSYA could significantly increase HO-2 expression, adenosine triphosphate (ATP) level, and Mn-superoxide dismutase (SOD) activity and restrain cytochrome c (cyto c) release and MDA level in H9C2 cells suffered from H/R, and this cardioprotective property of HSYA was mainly mediated via the phosphoinositide 3-kinase (PI3K)/Akt/HO-2 pathway independent of extracellular regulated protein kinases (ERK)/glycogen synthase kinase-3*β* (GSK-3*β*) pathway.

The toll-like receptor 4 (TLR4) signaling pathway may also take part in the protective effect of HSYA. Administration of HSYA inhibited the elevated expression of TLR4 as well as the increased indexes including infarct size, CK-MB, and LDH activity caused by MI/R. Further evidence was that HYSA failed to lessen MI/R damage in the TLR4-knockout mice. Additionally, in neonatal rat ventricular myocytes (NRVMs) subjected to H/R and lipopolysaccharide (LPS), HSYA increased cell viability and downregulated excessive tumor necrosis factor *α* (TNF-*α*) and interleukin-1*β* (IL-1*β*) and overexpressions of TLR4 and nuclear factor-kappa B (NF-*κ*B) [17].

#### 2.1.3. Antihypertension Effect

Hydroxysafflor yellow A offered the potential for reducing blood pressure. The mean arterial pressure (mAP) and HR (heart rates) in both of the normotensive rats and the spontaneously hypertensive rats (SHR) could be markedly reduced by HSYA [18]. Further study disclosed that HSYA could downregulate the levels of left ventricular systolic pressure (LVSP), left ventricular end-diastolic pressure (LVEDP), and the maximum rate of increase of left ventricular pressure (+d*p*/d*t*_max_) as well as HR but had little effect on the maximum rate of decrease of left ventricular pressure (-d*p*/d*t*_max_) in the isolated rat heart [18]. Mechanistically, large conductance Ca^2+^-activated K^+^ channel (BK_Ca_) and ATP-sensitive potassium channel (K_ATP_) was responsible for the effects of HYSA on blood pressure and cardiac function [18]. The test* in vitro* showed that HSYA could enhance the reduced diastolic response induced by acetylcholine (Ach) and sodium nitroprusside (SNP) and attenuate the vascular contractile effect of PE in the aorta ring isolated from the model [19]. In rat thoracic aorta rings, HSYA inhibited PE-induced endothelium-independent vasoactive response via inhibiting inositol 1,4,5-triphosphate (IP3) receptor in VSMCs and thus reducing extracellular Ca^2+^ influx and intracellular Ca^2+^ release [20].

Vascular adventitia proliferation and hyperplasia are of great importance for hypertension occurrence. HSYA has a suppressive effect on rat adventitial fibroblasts proliferation and collagen synthesis stimulated by angiotensin II (Ang II)* in vitro*. It also inhibited the elevated expressions of matrix metallopeptidase-1 (MMP-1), TGF-*β*1, *α*-smooth muscle actin (*α*-SMA), and NF-*κ*B p65 in this model [21]. HYSA inhibited proliferation and dedifferentiation of aorta vascular smooth muscle cells (VSMCs) exposed to platelet-derived growth factor- (PDGF-) BB into a proliferative phenotype, which might be associated with its inhibition of nitrous oxide (NO) and cyclic guanosine monophosphate (cGMP) production, Akt signaling activation, and cycle related proteins as well as its elevation of HO-1 in VSMCs [22]. At the same time, HYSA could suppress proliferation and migration of VSMCs induced by LPS and downregulate levels of TNF-*α* and interleukin-6 (IL-6) as well as interleukin-8 (IL-8) via the TLR-4/ ras-related C3 botulinum toxin substrate 1 (Rac1)/Akt pathway [23].

HSYA could also act on pulmonary artery. It dose-dependently blocked the progression of pulmonary artery remodeling, decreased the cell count in the small pulmonary bronchioles, attenuated right ventricular hypertrophy, and reduced mean right ventricular systolic pressure (mRVSP) in the pulmonary arterial hypertension (PAH) rats induced by hypoxic [24]. In addition, HSYA exerted a vasorelaxing effect on phenylephrine- (PE-) stimulated vascular constrictive action in rat pulmonary artery (PA) rings in a concentration-dependent manner via Kv activation of pulmonary artery smooth muscle cells (PASMCs) [25]. Further study demonstrated that HYSA could reduce pulmonary arterial hypertension via increasing SOD activity and decreasing levels of MDA and 8-hydroxydeoxyguanosine (8-OHdG) and mRNA of IL-1*β*, IL-6, and TNF-*α* [26].

#### 2.1.4. Effect on Cardiac Hypertrophy

In the overload-induced cardiac hypertrophy rats, HSYA exhibited significantly ameliorative effect on left ventricular mass index (LVMI) induced by the ligation of abdominal aorta, as a consequence of the alleviation of pathological lesion, including smaller cardiac muscle fibers and lightly stained cardiomyocytes nuclei [27]. Additionally, HSYA treatment inhibited cell apoptosis by upregulating Bcl-2/Bax ratio and decreasing matrix metallopeptidase-2 (MMP-2) and MMP-9 levels in serum [27].

### 2.2. Neuroprotective Effects

HYSA offered the therapeutic potential for being natural sources on brain diseases including cerebral ischemia, dementia, Parkinson's disease (PD), and traumatic brain injury (TBI). Previous studies* in vivo* and* in vitro* provided further support and evidence for the neurological use of HYSA.

#### 2.2.1. Effect on Cerebral Ischemia

HYSA is considered as a protective agent against cerebral ischemia, which is now a hot research topic of modern medicine. Boring the similar potency to nimodipine, HSYA was found to exert significant neuroprotective effects on the permanent middle cerebral artery occlusion (MCAO) induced focal cerebral ischemic rats as expressed by the reduced neurological deficit scores, infarct area, edema extend, and cell apoptosis [28–30]. The anticerebral ischemic effect of HYSA might result from its suppression of platelet aggregation, thrombin generation, cerebrovascular contraction, cerebrovascular permeability, and thrombin-mediated inflammation as well as promotion of prostacyclin (PGI2)/thromboxane (TXA2) ratio and hemorheology. HYSA could also decrease Ang II, resulting in NF-*κ*B p65 nuclear translation, p65 binding activity, elevation of ICAM-1 mRNA and protein levels, and neutrophils infiltration [6, 31, 32].

The neuroprotective actions of HSYA* in vivo* resulted from several reasons. Chen* et al.* reported that HSYA critically decreased the apoptosis cell number and increased the Bcl-2/Bax proportion in the penumbral cortex of rats subjected to the transient MCAO for 2 h and followed by 24 h reperfusion by the PI3K/Akt/GSK-3*β* pathway [33]. Also, HSYA treatment could cause an increase in the level of brain-derived neurotrophic factor (BDNF) in the MCAO mice due to inhibiting the TLR4 signaling pathway [34]. A study by Qi* et al.* [35] revealed that this effect could be blocked by an Akt inhibitor. The further study indicated that the promotion of HSYA on Akt-autophagy pathway occurred in neuronal-specific cells of penumbra tissue. Additionally, it was found that HYSA inhibited NF-*κ*B pathway and restored the metabolism pathways in the MCAO rats [36].

Blood-brain barrier (BBB), the main shield between cerebral capillaries and brain parenchyma for delivering therapeutic compounds into the brain, shows close relationship with brain ischemic damage. HYSA could penetrate across BBB, downregulate expressions of 12/15-lipoxygenase (12/15-LOX) and its metabolite, and trigger decrease of BBB permeability and improvement of tight junction in the MCAO mice via attenuating of occludin, claudin-5, and ZO-1 expressions as well as regulating the tight junction pathway [37–39].

*In vitro*, HYSA inhibited neurons injury stimulated by glutamate, sodium cyanide (NaCN), and OGD by preventing cell death and LDH release in cultured rat fetal cortical cells [28, 29]. Additionally, HSYA alleviated tyrosine nitration induced by authentic peroxynitrite in bovine serum albumin and primary cortical neurons [40]. The further study showed HSYA protected PC12 cells from OGD-induced apoptosis followed by reperfusion through suppressing intracellular oxidative stress and mitochondrial-mediated pathway [41]. In the LPS-activated coexistence system for microglia and neurons, HYSA activated microglia by suppressing TLR4 expression earlier, resulting in later appearance of neuronal apoptosis. The later study showed that the TLR4 pathway played a role in the protective effect of HYSA [42]. Moreover, in cerebral ischemia, release of excessive glutamate always leads to* N*-methyl-D-aspartate receptors (NMDARs) overactivation and excitotoxic neuron injury. Without any effect on the expressions of NR2B-containing NMDARs, HSYA protected rat cortical neurons subjected to* N*-methyl-d-aspartate (NMDA) from cell apoptosis via decreasing Bax expression, increasing Bcl-2 expression and downregulating expressions of NR2B-containing NMDARs instead of NR2A-containing NMDARs [43]. Wang* et al*. further elucidated that HSYA concentration-dependently inhibited excitatory postsynaptic currents (EPSCs) mediated by NMDARs and increased P2/P1 ratio (PPR) of both NMDAR EPSCs and a-amino-3-hydroxy-5-methyl-4-isoxazolepropionic acid receptor (AMPAR) EPSCs resulting in suppressing release of presynaptic transmitter in the mouse hippocampus CA1 region, with a mechanism that HSYA inhibited the membrane depolarization current magnitude induced by NMDARs and ischemic long-term-potentiation (LTP) stimulated by OGD [44]. Moreover, the NMDA-mediated and NMDAR-induced intracellular Ca^2+^ influx, the NMDAR-induced cell apoptosis and necrotic cell death, and the NMDA-induced mitochondrial injury were inhibited by HYSA in hippocampal neurons [44].

Notably, mitochondria are also likely to be involved in the underlying mechanism. In the cortex mitochondria of rats, HYSA ameliorated the damage induced by cerebral ischemia by inhibiting overloaded Ca^2+^ and scavenge capability of free radicals, increasing the membrane fluidity and the activities of respiratory enzymes and decreasing the edema degree and the membrane phospholipid decomposability [45]. However, this protective effect would be attenuated by inhibition of the opening of MPTP [46]. And the* in vitro* study found out that Ca^2+^- and H_2_O_2_-stimulated swelling of mitochondria isolated from rat brains was inhibited by HYSA. Meanwhile, HYSA reduced Ca^2+^ overload-induced ROS generation, improved mitochondrial energy metabolism, and increased ATP level and the respiratory control ratio [47].

#### 2.2.2. Effect on Dementia

Recent findings have discovered the antidementia property of HYSA and provided a foundation for its clinical use in both of vascular dementia (VD) and Alzheimer's disease (AD). HSYA could improve spatial learning and memory in the rat model of VD via upregulating the expressions of VEGF-A, N-methyl-D-aspartic acid receptor 1 (NR1), BDNF, and NMDAR in the hippocampal, which enhanced LTP and increased synaptic plasticity consequently [48, 49]. HSYA could significantly reverse cognitive impairment induced by homocysteine (Hcy) in rats, and this was related to attenuation of A*β*_40_ and A*β*_42_ levels in hippocampus partially via suppressing PS1 protein level, rescuing apoptosis, and increasing LTP in the AD model [50]. Zhang* et al*. implied that the protective effect of HYSA on the A*β*_1-42_-induced AD mice could be explained by inhibition of inflammation via enhancing the phosphorylation of JAK2/STAT3 pathway [51, 52]. HYSA also displayed a protective effect from neurotoxicity induced by A*β*_25-35_ in rat pheochromocytoma (PC12) cells by increasing cell viability, stabilizing mitochondrial function, and inhibiting oxidative stress characterized by reduced levels of lactate LDH, intracellular ROS and MDA, and neuronal apoptosis [53].

#### 2.2.3. Effect on Parkinson's Disease

HYSA is also a potential candidate drug for PD, a long-term degenerative disorder. This agent had a neuroprotective role in the 6-hydroxydopamine- (6-OHDA-) induced PD rats. It could also increase the levels of dopamine and its metabolites, glial cell line-derived neurotrophic factor (GDNF) and brain-derived neurotrophic factor (BDNF) in striatum of PD rats [58]. Meanwhile, HSYA could effectively relieve motor dysfunction of the PD mice model induced by rotenone and protect dopamine neurons by elevating TH-containing dopaminergic neurons and dopamine content in the striatum in the PD mice. It was suggested that BDNF/tyrosine kinase receptor type B (TrkB)/dopamine receptor 3 (DRD3) signaling pathway was responsible for the pharmacological effect of HSYA [59]. Additionally, after coadministration of HSYA with L-DOPA, the 6-OHDA-induced PD rat model exerted the attenuated dyskinesia, the prolonged motor response duration, and the downregulated expression of dopamine D receptor in the striatum, compared with the PD rats administrated by L-DOPA only [60]. Besides, it was demonstrated that HSYA improved cell viability, reduced cell apoptosis, and increased levels of SOD and glutathione (GSH), the ratio of Bcl-2/Bax, and mRNA levels of neuron-specific enolase (NSE) and microtubule-associated protein-2 (MAP-2), providing a mechanism of HYSA protecting the differentiation of mesenchymal stem cells (MSCs) against *β*-mercaptoethanol (BME) causes oxidation [61].

#### 2.2.4. Effect on Traumatic Brain Injury

The activities of SOD and catalase (CAT), the level of GSH, and the GSH/oxidized glutathione (GSSG) ratio were enhanced while the levels of MDA and GSSG were reduced in the brain of the TBI rats after HSYA treatment [54]. Another report confirmed that HYSA increased activities of mitochondrial ATPase and tissue plasminogen activator (t-PA) and decreased plasma plasminogen activator inhibitor-1 (PAI-1) activity and MMP-9 expression in the hippocampus of the TBI rats [55].

#### 2.2.5. Effect on Other Nervous System Diseases

HSYA treatment markedly alleviated lymphostatic encephalopathy- (LE-) induced brain injury in rats by dramatically decreasing the neurological scores, attenuating histological changes especially cell apoptosis in the rostral ventrolateral medulla (RVLM) and reducing heart rate variability. Additionally, downregulation of endothelial nitric oxide synthase (eNOS) expression in both of mRNA and protein levels of the RVLM in LE rats were prevented by HYSA [56]. In the spinal cord compression injury rats, HSYA treatment significantly attenuated spinal cord edema and improved motor function outcomes in rats, and the potential mechanism of this action was through ameliorating extent of oxidative stress and preventing release of proinflammatory molecules with inhibition of NF-*κ*B [57].

### 2.3. Hepatoprotective Effects

The hepatoprotective activities of HYSA have attracted the attention from the researchers. Recent evidences support HYSA as an antifibrotic agent in hepatic disorders.

#### 2.3.1. Effect on Hepatic Fibrosis

The hepatoprotective effects of HYSA are especially related to its antifibrotic and antioxidative actions. In respect of antihepatic fibrosis assay* in vivo*, HSYA treatment could not only reduce the serum levels of alanine aminotransferase (ALT), aspartate transaminase (AST), hyaluronan (HA), laminin (LN), and type III procollagen (PC III) as well as the hepatic levels of ROS and MDA but also elevate the activity and mRNA of SOD, glutathione peroxidase (GPx), and expression of TGF-*β*1 in liver tissue of the long-term alcohol-injured rats [62]. The histological studies suggested the alcohol induced liver damage such as hepatic fibrogenesis which could be greatly alleviated by HYSA [62]. Also, HYSA could decrease levels of total cholesterol (TC), triglyceride (TG), and mRNA expressions of transforming growth factor *β* receptor I (TGF*β*-R I), transforming growth factor *β* receptor (TGF*β*-R II), mitogen-activated protein (MAP), ERK, MAP/ERK kinase kinase 3 (MEKK3), and MAP kinase kinase-5 (MEK5) as well as phosphorylation of ERK5 in the tetrachloride- (CCl_4_-) induced hepatic fibrosis rats [63, 64]. The precise mechanism of the hepatoprotective property was that HSYA strengthened expressions of peroxisome proliferator-activated receptor-*γ* (PPAR*γ*) and MMP-2, downregulated expressions of TGF-*β*1 and TIMP-1, and reduced *α*-SMA level by stimulating PPAR*γ* activity [65]. Meanwhile, the ameliorative effects of HYSA on the CCl_4_/ high fat diet- (HFD-) stimulated liver fibrosis rats were significantly alleviated by the PPAR*γ* inhibitor, which was a result of blocking p38 MAPK phosphorylation [66]. Hepatic stellate cells (HSCs) appear to be vital in the development of liver fibrosis [67]. The testing* in vitro* suggested proliferation of HSCs stimulated by H_2_O_2_ was inhibited by HSYA because of HSYA's blockage of the cell cycle from G_0_/G_1_ to G_1_/S [65]. Moreover, in HSCs, HYSA also inhibited cell proliferation and induced cell apoptosis in a dose- and time-dependent fashion accompanying with decreasing expressions of type I alpha collagen (Col I *α*), *α*-SMA and type III alpha collagen (Col III *α*), Bcl-2, and myocyte enhancer factor 2C (MEF2C) and increasing expressions of cyto c, Caspase-9, and Caspase-3, which resulted from the activation of the extracellular regulated protein kinases (ERK5) pathway as well as the extracellular regulated protein kinases 1/2 (ERK1/2) pathway [68, 69].

#### 2.3.2. Effect on Hepatic Ischemia

HSYA reduced serum AST and ALT levels and expressions of TNF-*α* and IL-1*β*, ameliorated inflammation and necrosis, and blocked macrophage recruitments in the I/R mice. Similarly,* in vitro*, pretreatment of HYSA resulted in the weakened migratory reaction and the reduced inflammatory cytokines in the H/R-challenged RAW264.7. The further study showed that HSYA had suppressive effect on expressions of MMP-9 and ROS, NF-*κ*B activation, and p38 phosphorylation in the injured RAW264.7 cells [70].

### 2.4. Pulmonary Protective Effect

The various researches exhibited remarkable pulmonary protective properties of HYSA* in vivo* and* in vitro*, especially about the inhibitory effect on chronic obstructive pulmonary disease (COPD), acute lung injury (ALI), and lung fibrosis.

#### 2.4.1. Effect on Airway Inflammation

HSYA treatment could attenuate the airway hyperresponsiveness (AHR) cell chemotaxis, proinflammatory cytokines, type 2 helper T cell 2 (Th2) cytokines, total and ovalbumin- (OVA-) specific IgE and adhesion molecules in bronchoalveolar lavage fluid (BALF), and inflammatory responses levels in the lung tissue of the OVA-induced asthmatic mice. Study suggested that HSYA treatment inhibited the transcriptional activity of NF-*κ*B by inhibiting NF-*κ*B p65 nuclear translocation and nuclear factor of I*κ*B-*α*phosphorylation and degradation [71]. Wang* et al*. reported that HSYA markedly suppressed the thickening and collagen deposition of the small airway and weakened TGF-*β*1 mRNA and protein expression as well as type I collagen (Col I) and *α*-SMA expressions in the lung of the COPD model in the rats stimulated by cigarette smoke and LPS. In addition, HSYA elicited inhibitory effect on phosphorylation of p38 mitogen-activated protein kinase (MAPK) in the rat lung tissue [72].

#### 2.4.2. Effect on Acute Lung Injury

HSYA could alleviate pulmonary edema, reduce acidosis, increase partial pressure of oxygen (PaO_2_), and inhibit inflammatory cell infiltration, lung mRNA expressions of TNF-*α* and ICAM-1, and levels of plasma IL-6 and IL-1*β* [73]. HSYA significantly increased the activities of antioxidant enzymes, inhibited the inflammatory response via the cAMP/PKA pathway activation, and attenuated OA-induced lung injury [74]. Additionally, HSYA decreased lung permeability, platelet count, and ADP-mediated platelet aggregation as well as cytokine levels in serum and BALF in the aged rats with gasoline engine-induced lung injury. Moreover, it suppressed overexpressions of ICAM-1, vascular cell adhesion molecule-1 (VCAM-1), and proinflammatory cytokines in platelets and lung tissue. And decrease in cAMP level in lung and platelets and PKA activity and PPAR*γ* expression in platelets induced by gasoline engine exhaust were also reversed by HYSA [75]. Meanwhile, in the sepsis-associated ALI mice, the inflammatory infiltration and proinflammatory cytokine expressions in lung, pneumochysis, and respiratory insufficiency induced by LPS were greatly relieved by HYSA treatment. Its suppression of p38MAPK phosphorylation as well as NF-*κ*B activation was responsible for this protective effect [76]. Also, HSYA ameliorated the pathological state and lung vascular permeability of the LPS-induced ALI in mice. This effect was accompanied with the negative effect on pulmonary myeloperoxidase (MPO) activity and levels of serum TNF-*α*, IL-1*β*, IL-6, and interferon-*β* (IFN-*β*). The further investigation demonstrated that intraperitoneal injection of LPS to the mice resulted in upregulation of protein expressions of TLR4, myeloid differentiation factor 88 (MyD88) and Toll/IL-1 receptor- (TIR-) domain-containing adapter-inducing interferon-*β* (TRIF) and phosphorylation of MAPKs, translocation of NF-*κ*B/p65, and downregulation of I*κ*B-*α*, all of which could be deteriorated by HSYA [77]. Zhang* et al.* observed the negative influence of HSYA on the binding of LPS to the cell membrane receptor in the LPS-caused ARDS mice model [78]. And injection of HYSA alleviated the mRNA and protein levels of Col I, type III collagen (Col III), *α*-SMA, myeloid differentiation-2 (MD-2), and cluster of differentiation 14 (CD14) as well as inflammatory factors in plasma or lung and the collagen deposition in lung via suppressing the TLR4/NF-*κ*B pathway. The* in vivo* test demonstrated the inhibitory effect of HYSA on the specific binding of LPS to receptors on A549 or Eahy926 cell membranes, suggesting the TLR4 receptor a target of HSYA on the cell membrane [78].

#### 2.4.3. Effect on Lung Fibrosis

Injection of HYSA attenuated the pathologic changes of pulmonary inflammation, increased the body weight, PaO_2_ and decreased PaCO_2_, mRNA expressions of TNF-*α*, IL-1*β*, TGF-*β*1, and IL-6, MDA activity, and the count of NF-*κ*B p65 positive cells in the BLM-injured acute inflammation rats. This protective effect of HYSA might be due to inhibition of NF-*κ*B activation and p38 MAPK phosphorylation in lung tissue [79, 80]. Moreover, 21 days of HSYA to the BLM-induced chronic pulmonary fibrosis rats resulted in fibrosis amelioration by decreasing collagen deposition, and mRNA expression of TGF-*β*1, *α*-SMA and Col I connective tissue growth factor (CTGF) as well as *α*-SMA level [80, 81].

Assayed with human alveolar epithelial A549 cells, HYSA was found to inhibit Smad3 phosphorylation and Col I mRNA expression stimulated by TGF-*β*1 [81]. And HSYA exhibited inhibitory effects on LPS-induced inflammatory response in A549 cells by suppressing myeloid differentiation factor 88 (MyD88), TLR-4, TNF-*α*, ICAM-1, IL-1*β*, and IL-6 at the mRNA and protein expression levels and leukocytes adhesion to A549 cells. The mechanism was due to its negative regulation of NF-*κ*B p65 nuclear translocation and p38 MAPK phosphorylation [82]. Meanwhile, TGF-*β*1-induced alteration in proliferation, migration, extracellular matrix (ECM) accumulation, and degradation of human fetal lung fibroblasts MRC-5 were inhibited by HYSA. Further study disclosed that HSYA blocked the binding of TGF-*β*1 to the cytoplasmic receptors of TGF-*β*1-stimulated MRC-5 including TGF*β-*R II and suppressed the lung pathological alteration and *α*-SMA, Col I*α*1, and FN expressions as well as phosphorylation of mothers against decapentaplegic homolog 2 (Smad2), mothers against decapentaplegic homolog 3 (Smad3) and ERK, nuclear translocation of Smad2 and Smad3, and the binding of Smad3 to Col I promoter [83, 84].

### 2.5. Antitumor Effects

Several reports have provided strong evidences that HYSA is valuable for oncotherapy. HYSA was considered as a natural compound which could inhibit multistage carcinogenesis processes such as progression, adhesion, invasion, and migration. In HYSA-treated MCF-7 cells, Li* et al*. [85] observed increased cell apoptosis and ROS level, upregulated expressions of Bax and p53, blocked cell cycle, downregulated Bcl-2 and cyclin D1, released cyto c, activated Caspase-3, and disrupted MPP with the mechanism of its negative regulation on the NF-*κ*B/p65 nuclear translocation. Also, HSYA, acting as a PPAR*γ* agonist, showed inhibitory effect on proliferation and cell cycle transition and stimulatory effect on cell apoptosis in BGC-823 cells [86]. As for abnormally proliferated HUVECs cultured in HepG2 cell cultural supernatant, HSYA suppressed the expressions of vascular endothelial growth factor (VEGF) and its kinase insert domain receptor (KDR) through the Ras-Raf-MEK-ERK1/2 signaling pathway and dephosphorylated the corresponding kinase molecules [87]. Moreover, HYSA exerted inhibitory effect on cancer cell growth of tumor-bearing mice. This effect was associated with its downregulation of angiogenesis. Xi* et al*. [88] proved that it reduced the microvessel count and microvessel density of the transplanted tumors in the BGC-823 cell bearing mice while Yang* et al*. [89] reported this drug had the advantage of decreasing VEGF-A, basic fibroblast growth factor (bFGF), vascular endothelial growth factor receptor 1 (VEGFR1), and mRNA expression levels of cyclin D1, c-myc, and c-fos in the BGC-823 tumor-bearing mice through suppressing the ERK/MAPK and the NF-*κ*B pathway. Additionally, along with the ability of inhibiting the process of SMMC-7721 covering proliferation, adhesion, invasion, and migration, HYSA could suppress pulmonary metastasis of liver cancer. In the pulmonary metastatic mouse model of H22 cells, the formation of a complex with E-cadherin/*β*-catenin induced by HYSA could activate the expression of PPAR*γ* and inhibit the activity of MMP-2, finally leading to decrease in degradation of ECM and suppression of epithelial-mesenchymal transition (EMT) [90]. In addition, HSYA could reversibly and noncompetitively inhibit of human recombinant aldehyde dehydrogenase 1 (ALDH1) (Ki = 0.267 ± 0.024 mM) indicating it as a potential agent for treating ALDH1-associated cancers [91].

### 2.6. Metabolic Regulation Effect

The quantity of preadipocytes, adipocytes differentiation, and lipid accumulation play a key role in lipid metabolism [92]. HSYA significantly time- and dose-dependently prohibited the proliferation of 3T3-L1 cells. This effect was accompanied by a decline in the amount of intracellular lipid and triglyceride (TG) and a rise in mRNA expression of hormone-sensitive lipase (HSL) and promoter activities [93]. HSYA competitively inhibited *α*-glucosidase in a reversible way with* IC*_50_ = 1.1 ± 0.22 mM and Ki=1.04 ± 0.23mM, respectively. HSYA-induced structural change of *α*-glucosidase was mainly regional unfolding [94]. Advanced glycation end products (AGEs) and methylglyoxal (MGO) accumulation usually appear in individuals with diabetes and cause the occurrence of vascular complication [95]. Ni* et al*. [96] demonstrated that MGO-induced bovine serum albumin (BSA) glycation could be inhibited by HYSA. Besides, HSYA showed significantly inhibitory effect on glucose- (GLU-) induced development of AGEs formation and* N*-acetyl-glycyl-lysine methyl Ester (G.K.) peptide-mediated ribose glycation. Also, HSYA could defend against MGO-induced damage in cultured human brain microvascular endothelial cells (HBMEC) by increasing cell viability and decreasing cell mortality. It suppressed cell apoptosis and Caspase-3 expression in HBMEC and inhibited AGEs accumulation in HBMEC after treated with MGO [97].

### 2.7. Endothelium Cell Protection

*In vivo*, HSYA effectively recovered perfusion of ischemic hindlimb tissue and gave a rise of the arteriole and capillary densities in the femoral artery-interrupted ischemic gastrocnemius muscles of the mice [98]. Ox-LDL-caused endothelial injury could be relieved by HSYA dose-dependently. Proteomic investigation revealed that this effect was related to the antiapoptotic activity of voltage dependent anion-selective channel 2 (VDAC2) [99]. Moreover, HSYA promoted the viability of LPS-injured HUVEC. It also inhibited the subsequent inflammation induced by LPS in HUVEC including NF-*κ*B p65 subunit DNA binding, I*κ*B*α* phosphorylation, ICAM-1, and E-selectin mRNA levels elevation and phosphorylation of p38 MAPK or c-Jun N-terminal kinase (JNK) MAPK, cell surface ICAM-1 protein expression, and leukocyte adhesion to HUVEC [100]. Meanwhile, HYSA elicited a protective effect on HUVECs from hypoxia by attenuating cell apoptosis and cell cycle arrest, which was the result of the upregulation of the Bcl-2/Bax ratio and the hypoxia inducible factor-1*α*- (HIF-1*α*-) VEGF pathway as well as NO contend and eNOS expression and downregulation of p53 protein expression [101]. However, with little effect on the normal HUVEC, HSYA caused an increase of the capillary-like tube formation and migration of HUVEC, which could be reversed by an anti-Tie-2 neutralizing antibody. Expression of angiopoietin 1 and phosphorylation of Tie2, Akt, and ERK 1/2 were greatly elevated by HSYA [102].

### 2.8. Other Effects

As an anti-inflammatory molecule, HYSA could inhibit LPS-induced NLRP3 inflammasome activation via binding to Xanthine Oxidase in mouse RAW264.7 macrophages [103]. HSYA-mediated sonodynamic therapy could induce ROS-dependent autophagic response via the PI3K/Akt/mTOR signaling pathway in THP-1 macrophages [104]. Besides, HSYA inhibited rabbit polymorphonuclear (PMN) activation induced by LPS by reducing LPS-induced elevated adhesion potency, free calcium concentration, TNF-*α* and IL-6 mRNA expression, and NF-*κ*B p65 nuclear translocation [105]. Also, HYSA exhibited anti-inflammatory effect as evidenced by inhibiting the IL-1*β*-induced release of IL-6, IL-8, and MMP-1 in SW982 human synovial cell, and this was associated with suppression of ERK, NF-*κ*B, and activator protein-1 (AP-1) signaling [106]. And HSYA could reduce strike-triggered local oedema and neutrophil infiltration in skeletal muscle via inhibiting p38 MAPK phosphorylation and suppressing NF-*κ*B pathway activation [107]. It was also an effective therapeutic agent in ameliorating sepsis-induced apoptosis of cluster of differentiation 4+ (CD4+) T lymphocytes through its anti-inflammatory and antiapoptotic effects [108].

HSYA had protective effect against fibrosis in renal cells, through inhibiting TGF-*β*1/Smad3-mediated epithelial-mesenchymal transition signaling pathway [109]. Topical use of HSYA could relieve the UV-induced skin damage in mice by promoting recovery from stretching, inhibiting epidermal hyperproliferation and keeping the structural integrity of the skin via antioxidative activity [110]. Moreover, HSYA could strongly inhibit tyrosinase by binding and changing the tertiary structure of tyrosinase [111].

## 3. Conclusion

**Figure 2:**
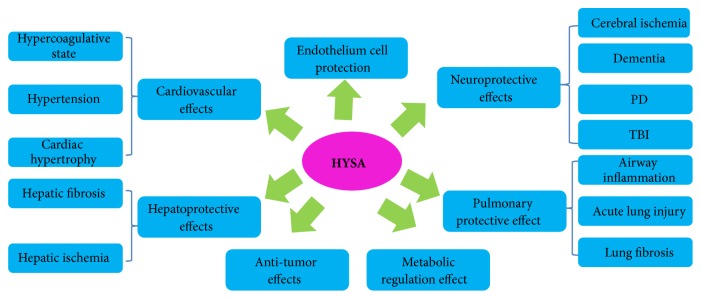
Pharmacological effects of HYSA.

**Figure 3:**
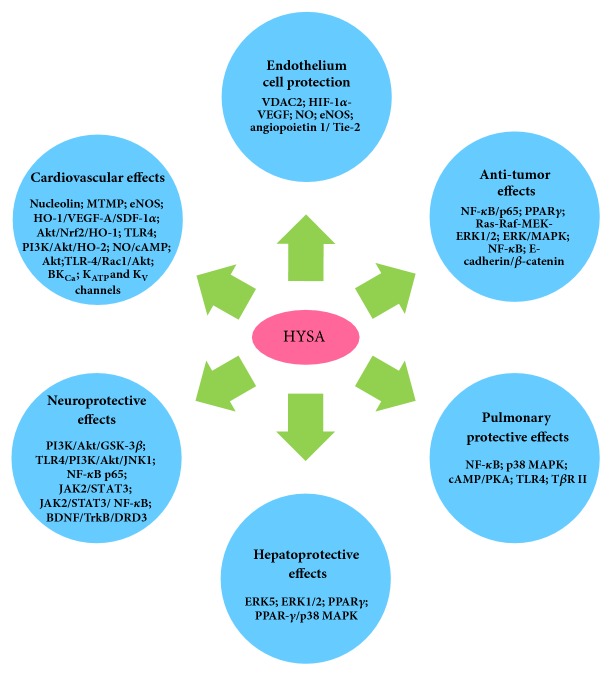
Action mechanism and corresponding mediators of HYSA.

Noncommunicable diseases (NCDs), namely, heart disease, stroke, cancer, diabetes, and chronic lung disease, are causing worldwide public health problem and alarmingly leading causes of almost 70% deaths. Natural chemicals are favorable resources that may be utilized to develop such agents. HYSA, a component isolated from safflower with little toxicity [112], showed various pharmacological effects* in vitro *and* in vivo *(summarized in Figure 2), including cardiovascular protection (summarized in Table 1), neuroprotection (summarized in Table 2), metabolism regulation, antitumor effect, and liver, lung, and EC protective activities. These activities indicate the use of HYSA for prevention and/or the treatment of NCDs and other intractable diseases such as AD, PD, TBI, ALI, and fibrotic diseases. Although HYSA possesses extensive pharmacological effects, it is currently used as a drug mainly in treating cardiovascular and cerebrovascular diseases. It needs to be clinically explored in other aspects especially for respiratory, hepatic and metabolic diseases, and malignancy. Recent studies focusing on various bioactivities of HYSA have yielded promising results, demonstrating both the pharmacological effects and the molecular mechanisms of HYSA (summarized in Figure 3). Further investigation of the molecular mechanisms of HYSA is anticipated to expand the clinical applications of HYSA.

## Figures and Tables

**Table 1:** Summary about the cardiovascular protection of HYSA.

Effects	Model	Details	Ref.
Anti-coagulant effect	Platelets exposed to ADP	Inhibiting aggregation.	[6]
	Rats	Inhibiting thrombosis formation.	[6]
	Blood sample from rabbits	Inhibiting platelet aggregation induced by ADP and PAF and decreasing blood viscosity.	[6]
	Blood sample from rabbits	Inhibiting WRP aggregation and PMNs aggregation induced by PAF	[7]
	Rabbit plasma	Prolonging PT and RT.	[8]

Myocardial ischemia	LAD-induced MI mice	Reversing the haemodynamic changes including LVSP, +*d*p/*d*t and −*d*p/*d*t, enhancing the survival rate, increasing expressions of nucleolin and VEGF-A.	[9]
	LAD-induced MI rats	Reducing CK-MB, ROS, cTnI and 8-OHdG expression, and increasing SOD activity.	[10]
	H9C2 cells subjected to OGD	Reducing CK-MB, LDH, MDA, ROS and cell apoptotic number and increasing MMP, SOD activity, and expressions of PGC-1*α* and Nrf2.	[11]
	ISO-induced MI rats	Reducing CK-MB, LDH, MDA, ROS and cell apoptotic number, inhibiting cell apoptosis and increasing MMP, SOD activity and expressions of PGC-1*α* and Nrf2.	[11]
	MI/R rats	Reducing the infarct size and the release of LDH.	[12]
	Ca^2+^-induced ventricular myocytes	Reducing mitochondria swelling, increasing phosphorylated eNOS protein and preventing cell death and depolarization of the mitochondrial membrane.	[12]
	Cardiac myocytes stimulated by anoxia and reoxygenation	Reducing cell viability and closed MPTP rods, and increasing opened MPTP round cells.	[13]
	Cardiac myocytes introduced by ionomycin	Increasing opened MPTP round cells and decreasing closed MPTP rods.	[13]
	EPCs stimulated by SDF-1*α*	Increasing CXCR4 expression.	[14]
	MI mice caused by the ligation of left coronary artery	Increasing EF, FS, EPC number, VEGF-A and SDF-1*α* and reducing apoptotic signals, TGF-*β* and Col I.	[14]
	H9C2 exposed to H/R	Reducing LDH, apoptosis index, Bcl-2/Bax ratio, cleaved Caspase-3 and increasing expression and activity of HO-1, Akt phosphorylation and Nrf2 translocation.	[10, 15]
	H9C2 exposed to H/R	Increasing cell viability, ATP, Mn-SOD and HO-2 and reducing cyto c, MDA, LDH, apoptosis index and Caspase-3.	[16]
	NRVMs subjected to H/R and LPS	Increasing cell viability and reducing levels of TNF-*α* and IL-1*β* and expressions of TLR4 and NF-*κ*B.	[17]
	MI/R rats accompanied hyperlipidemia trigged by high-fat diet	Reducing TLR4 expression, infarct size, CK-MB and LDH activity.	[17]

Artery constriction	SHR and the normotensive rats	Reducing mAP and HR *in vivo*, and down-regulating LVSP, LVEDP, +*d*p/*d*t_max_ and HR in the isolated rat heart.	[18]
	Immunized rats	Reducing systolic BP, HR, ET, ox-LDL and NO and relieving the histological change in the thoracic aortic endothelium.	[19]
	Aorta ring isolated from the immunized rats	Enhancing the diastolic response induced by Ach and SNP and attenuating the vascular contractile effect of PE.	[19]
	Rat thoracic aorta rings induced by PE	Inhibiting IP3 receptor in VSMCs and thus reducing extracellular Ca^2+^ influx and intracellular Ca^2+^ release.	[20]
	Ang II-stimulated rat adventitial fibroblasts	Inhibiting proliferation and collagen synthesis and decreasing expressions of MMP-1, TGF-*β*1, *α*-SMA and NF-*κ*B p65.	[21]
	VSMCs exposed to PDGF-BB	Reducing cell viability, cyclin D1, cyclin E, CDK2, CDK4, cGMP level and NO content and inhibiting phenotype switching and increasing HO-1 expression.	[22]
	VSMCs induced by LPS	Inhibiting proliferation and migration and down-regulating levels of TNF-*α*, IL-6 and IL-8.	[23]
	PASMCs under hypoxia	Inhibiting proliferation and cell cycle.	[24]
	PAH rats induced by hypoxic	Blocking the progression of pulmonary artery remodeling, decreasing the cell count in the small pulmonary bronchioles, attenuating right ventricular hypertrophy and decreasing mRVSP and protein expressions of PCNA and Ki67.	[24]
	PE-induced rat PA	Showing vasorelaxing effect independent of PA endothelium.	[25]
	PAH rats stimulated by MCT	Reducing RVSP, mPAP, RV/LVPS, the mean percent wall thickness of pulmonary arterioles and the vascular muscularization and increasing expressions of IL-1*β*, IL-6 and TNF-*α* in the mRNA level and SOD activity and decreasing levels of MDA and 8-OHdG.	[26]

Cardiac hypertrophy	Overload-induced cardiac hypertrophy rats induced by the ligation of abdominal aorta	Reducing LVMI, inhibiting cell apoptosis by up-regulating Bcl-2/Bax ratio and decreasing expressions of MMP-2 and MMP-9.	[27]

**Table 2:** Summary about the neuroprotection of HYSA.

Effects	Model	Details	Ref.
Cerebral ischemia	Rat fetal cortical cells stimulated by OGD.	Preventing cell death and LDH release.	[28]
	Rat fetal cortical cells stimulated by glutamate and NaCN	Preventing cell death and release of LDH and NO.	[29]
	MCAO rats	Reducing neurological deficit scores, infarct area, edema extend, cell apoptosis and expressions of Bax, Caspase-3, ICAM-1, IL-1*β*, TNF*-α *and NF-*κ*B, improving glucose metabolism and increasing expressions of GFAP, NGF and Bcl-2.	[28–30]
	MCAO rats	Decreasing infarct sizes, neurological deficit scores and cerebrovascular permeability.	[6]
	Coronary artery and basilar artery rings of dogs	Having a stronger effect on cerebrovascular vasodilatation than on cardiovascular vasodilatation.	[6]
	MCAO rats	Reducing neurological deficit scores, suppressing thrombin generation, suppressing thrombin generation, NF-*κ*B p65 nuclear translation, p65 binding activity, elevating ICAM-1 mRNA and protein levels and neutrophils infiltration and decreasing Ang II.	[31, 32]
	MCAO rats	Decreasing apoptotic cell number, increasing Bcl-2/Bax proportion, and elevating levels of Akt and GSK-3*β* phosphorylation in the penumbral cortex.	[33]
	MCAO rats	Decreasing infarct volume, and increasing BDNF and decreasing TLR4 expression, phosphorylation of NF-*κ*B p65, ERK1/2, JNK and p38 and secretion of TNF-*α*, IL-1*β* and NO.	[34]
	MCAO rats	Reducing infarct volume, improving neurological scores and activating Akt-autophagy.	[35]
	MCAO rats	Reducing infarct volume, neurological deficit scores, inhibiting expressions of TNF-*α*, IL-1 and IL-6, and p65 translocation and binding activity, up-regulating expression of IL-10 and restoring the metabolism pathways.	[36]
	MCAO rats	Decreasing BBB permeability, improving tight junction, attenuating expressions of occludin, claudin-5 and ZO-1 and regulating the tight junction pathway.	[37, 38]
	MCAO rats	Decreasing infarct volume, BBB permeability, brain edema, production of carbonyl and expressions of 12/15-LOX and 15-HETE and elevating nitrotyrosine.	[39]
	MCAO rats	Reducing infarct volume, BBB permeability, brain edema, expression of iNOS and level of NO end product and 3-NT and improving neurological scores.	[40]
	Bovine serum albumin and primary cortical neurons induced by authentic peroxynitrite	Alleviating tyrosine nitration.	[40]
	OGD-induced PC12 cells	Promoting cell viability, reducing MDA, apoptotic cells and expressions of Bax, Caspase-3 and cyto c and increasing Bcl-2 expression and SOD activity.	[41]
	LPS-activited co-existance system for microglia and neurons	Suppressing TLR4 expression, down-regulating MyD88, NF-*κ*B, JNK, ERK1/2 and p38 and release of TNF-*α*, IL-*β* and NO and increasing BDNF expression.	[42]
	Rat cortical neurons subjected to NMDA	Reducing cell apoptosis, expressions of Bax and NR2B-containing NMDA receptors and increasing Bcl-2 expression. Decreasing apoptotic cell number, increasing BCl_2_/Bax proportion, elevating levels of Akt and GSK-3*β* phosphorylation in the penumbral cortex.	[43]
	Mouse hippocampal slices	Inhibiting EPCs, postsynaptic NMDAR activity and pre-synaptic glutamate transmitter release, NMDAR-mediated OGD-evoked membrane depolarization current and NMDAR-dependent ischemic LTP induced by OGD.	[44]
	Mouse hippocampal neurons	Inhibiting NMDA-mediated and NMDAR- induced intracellular Ca^2+^ influx, NMDAR-induced cell apoptosis and necrotic cell deaths, and NMDA-induced mitochondrial injury.	[44]
	MCAO rats	Inhibiting overloaded Ca^2+^ and scavenge capability of free radicals, increasing the membrane fluidity and activities of respiratory enzymes and decreasing edema degree and membrane phospholipid decomposability in the cortex mitochondria.	[45]
	MCAO rats	Increasing latency time on rotarod and alteration behavior in Y-maze, levels of GSH and catalase, and reducing neurological deficit scores, levels of MDA and TNF-*β*, and infarct volume.	[46]
	Mitochondria isolated from rat brains suffered from Ca^2+^ and H_2_O_2_	Alleviating swelling, reducing ROS generation, improving mitochondrial energy metabolism and increasing ATP level and the respiratory control ratio.	[47]

Dementia	VD rats by 2-VO	Reducing escape latency, prolonged time spent in the platform quadrant and swimming distance in the water maze, and increasing LTP and finally ameliorating learning and memory, up-regulating expressions of VEGF-A, NR1, BDNF and NMDAR in the hippocampal.	[48, 49]
	AD rats induced by Hcy	Shortening escape latency, reducing the number to cross the hidden platform and time spent in the target quadrant, attenuating A*β*_40_, A*β*_42_ and PS1 protein levels, rescuing cell apoptosis and increasing LTP.	[50]
	A*β*_1-42_-induced AD mice	Decreasing memory deficits and expressions of Iba-1, GFAP, IL-1, TNF-*α* and iNOS, increasing expressions of IL-4 and IL-10 and suppressing activation of microglia and astrocytes in the hippocampi.	[51]
	A*β*_1-42_-induced BV-2 cells	Increasing cell viability, reducing mRNA levels of IL-1*β*, TNF-*α*, COX-2 and iNOS and protein expressions of COX-2, TNF-*α* and iNOS and up-regulating IL-4, IL-10 and phosphorylation protein expressions of JAK2 and STAT3.	[52]
	Neurons and SH-SY5Y cells inhibited by conditioned meadium of A*β*_1-42_-induced BV-2 cells	Promoting cell viability and inhibiting cell apoptosis.	[52]
	A*β*_25-35_-induced PC12 cells	Increasing cell viability, stabilizing mitochondrial function and reducing LDH, intracellular ROS, MDA and neuronal apoptosis.	[53]

TBI	TBI rats	Reducing contusion volume, MDA and GSSG in the brain, plasma PAI-1 activity and MMP-9 expression in the hippocampus, and increasing activities of SOD, CAT, GSH, t-PA and mitochondrial ATPase and the GSH/GSSG ratio.	[54, 55]

Other nervous system diseases	LE-induced brain injury in rats	Decreasing the neurological scores, cell apoptosis in RVLM, up-regulating eNOS expression in both of mRNA and protein levels of RVLM and reducing HRV.	[56]
	Spinal cord compression injury rats	Decreasing neurological deficit score, MDA, MPO, NO, TNF-*α*, IL-6, iNOS, COX-2, activities of NF-*κ*B and Caspase-3, water content in spinal cord and permeability in BSCB and increasing SOD activity.	[57]
